# EHMTI-0177. Evidence for plastic brain morphometric changes during the migraine cycle

**DOI:** 10.1186/1129-2377-15-S1-E10

**Published:** 2014-09-18

**Authors:** G Coppola, A Di Renzo, E Tinelli, E Iacovelli, C Lepre, C Di Lorenzo, G Di Lorenzo, D Di Lenola, V Parisi, F Pauri, G Fiermonte, F Bianco, F Pierelli

**Affiliations:** 1 Department of Neurophysiology of Vision and Neurophthalmology, G.B. Bietti Foundation-IRCCS, Roma, Italy; 2 Department of Neurology and Psychiatry Neuroradiology section, Sapienza University of Rome, Roma, Italy; 3 Department of medico-surgical sciences and biotechnologies Neurology section, Sapienza University of Rome, Roma, Italy; 4 Department of Neurology, Don Carlo Gnocchi Onlus Foundation, Milano, Italy; 5 Department of Systems Medicine, University of Rome ‘‘Tor Vergata’’ Laboratory of Psychophysiology Psychiatric Clinic, Roma, Italy; 6 Department of medico-surgical sciences and biotechnologies, Sapienza University of Rome Polo Pontino, Latina, Italy

## Background

Neurophysiological investigations have demonstrated that there are distinctive fluctuations in the brain’s electric signals between the ictal and interictal periods of recurrent migraine. Whether structural plasticity of the brain is also an important feature of episodic migraine remains unresolved.

## Aim

We therefore investigated the possibility that there are fluctuations over time in whole brain grey matter morphometry of patients affected by episodic migraine without aura (MO).

## Method

Twenty-four patients with untreated MO underwent MRI scans (3-Tesla Siemens Verio) during (n = 10) or between attacks (n = 14) and were compared to a group of 15 healthy volunteers (HV). We then performed voxel-based-morphometry (VBM) analysis of structural T1-weighted MRI scans to determine if changes in brain structure were observed over the course of the migraine cycle.

## Results

During the interictal phase, MO patients had a significantly lower grey matter (GM) density within the right inferior parietal lobule, right temporal inferior gyrus, right superior temporal gyrus, and left temporal pole than did HV. During attacks, GM density increased within the left temporal pole, bilateral insula, and right lenticular nuclei, but no areas exhibited decreased GM density.

## Conclusion

The morphometric GM changes between ictal and interictal phases reported in the present study suggest that abnormal structural plasticity may be an important mechanism of migraine pathology. Given the functional neuroanatomy of these areas, our findings suggest that migraine is a condition associated with global dysfunction of multisensory integration and memory processing.

No conflict of interest.

